# Merozoite surface protein-3α is a reliable marker for population genetic analysis of *Plasmodium vivax*

**DOI:** 10.1186/1475-2875-5-53

**Published:** 2006-07-03

**Authors:** Sedigheh Zakeri, Hesam Barjesteh, Navid D Djadid

**Affiliations:** 1 Malaria Research Group (MRG), Biotechnology Department, Pasteur Institute of Iran, Pasteur Avenue, P.O.BOX 1316943551, Tehran, Iran

## Abstract

**Background:**

The knowledge on population structure of the parasite isolates has contributed greatly to understanding the dynamics of the disease transmission for designing and evaluating malaria vaccines as well as for drug applications. *msp-1 *and *msp-3α *genes have been used as a genetic marker in population studies of *Plasmodium vivax *isolates. In this study, *msp-3α *was compared and assessed with *msp-1 *marker in order to find whether *msp-3α *is a reliable genetic marker for *P. vivax *population studies.

**Methods:**

This comparative study was designed and carried out as the first assessment of diversity in *Pvmsp-3α *gene by polymerase chain reaction-restriction fragment length polymorphism (PCR-RFLP) in the 50 northern and 94 southern *P. vivax *isolates from Iran, which had been analysed before for *msp-1 *gene.

**Results:**

Three allele size as, Type A (1.8 kb), Type B (1.5 kb) and Type C (1.2 kb) have been detected among both northern and southern isolates based on PCR results. Type C (70%) and Type A (68.7%) were the predominant fragments among northern and southern parasites, respectively. 99 distinct *Pvmsp*-3α fragments defined by the size were detected in the 94 southern samples by PCR analysis. However, no mixed genotype infections have been detected among northern isolates. Based on restriction pattern from digestion with *Hha I *and *Alu I *12 and 49 distinct allelic variants have been detected among 50 northern and 94 southern isolates. However, based *on msp-1 *gene, 30 distinct variants identified in all 146-sequenced Iranian *P. vivax *isolate.

**Conclusion:**

The results suggested that PCR-RFLP on *msp-3α *gene is an adequate, applicable and easily used technique for molecular epidemiology studies of *P. vivax *isolates without the need for further sequencing analysis.

## Background

Knowledge of the population structure of the parasite isolates is important to understand the dynamics of disease transmission. Molecular epidemiological studies require reliable polymorphic markers [1]. Four polymorphic markers have been used to study the genetic structure of *Plasmodium vivax*, including *Pvgam1*, which encodes for a protein expressed during sexual stages [2,3]*Pvcs*, the circumsporozoite protein [4-6], *Pvmsp-1 *coding for the merozoite surface protein 1 [7-11] and *Pvmsp-3α*, encoding for the merozoite surface proteins 3α [12-14].

A number of genes encoding for *P. vivax *MSPs have been identified, including *Pvmsp-1 *[8], *Pvmsp-3α*, *Pvmsp-3β *and *Pvmsp-3γ *[12,15], *Pvmsp-4 *and *Pvmsp-5 *[16] and *Pvmsp-9 *[17]. Of these, *Pvmsp-1 *has been most extensively studied and the sequence of the *Pvmsp-1 *gene varies between isolates from different part of the world. *Pvmsp-3α*, *Pvmsp-3β *and *Pvmsp-3γ *are members of a multi-gene family of related MSPs [8,15]. The three encoded proteins share only 35–38% identity and 48–53% similarity in pair-wise comparisons. They all contain similar structures including signal sequences and are expressed on the merozoite surface. The MSP-3α of *P. vivax *is a protein with a molecular weight ranging from 148 to 150 KD, an alanine-rich central domain and a series of heptad repeats that were predicted to form a coiled-coil tertiary peptide structure. *Pvmsp-3α*, like *Pvmsp-1*, is very polymorphic and has been used as a genetic marker in population studies of isolates from diverse geographic localities and origins [13,14,18].

In Iran, malaria is endemic in southeastern regions, including Hormozgan, Sistan and Baluchistan and the tropical part of Kerman provinces. In addition, malaria re-appeared in northern Iran in 1994 [19]. More than 80% of reported malaria cases in the south are due to *P. vivax *infection. Recent study on the 52 northern and 94 southern *P. vivax *parasite isolates carried out to determine the extent of genetic diversity and population structure of this parasite by using block 5 region of *Pvmsp-1 *gene [20]. A total of 7 and 27 distinct variants were detected among northern and southern isolates, respectively. Sequence alignments demonstrated the heterogeneity of *P. vivax *isolates in Iran and also showed that the parasites from the southern malaria endemic area were more polymorphic than those circulating in the northern area.

In this study, *msp-3α *was compared and assessed with the *msp-1 *marker in order to find out whether *msp-3α *is a reliable genetic marker for *P. vivax *population studies. Therefore, the same set of sequenced sample was examined here using the *Pvmsp-3α *gene by polymerase chain reaction-restriction fragment length polymorphism (PCR-RFLP).

## Materials and methods

### Study area and sample collection

*P. vivax *isolates were collected from two different malaria endemic areas as previously described [20]. This study was approved by the Ethical Review Committee of Research in Pasteur Institute of Iran. In this comparative study 50 northern and 94 southern isolates, which had been already sequenced for variable block 5 of *Pvmsp-1*, were selected for further analysis using *Pvmsp*-3α marker [20].

### Extraction of DNA

*P. vivax *genomic DNA was extracted from 250 μl of infected venous blood, using standard phenol/phenol-chloroform methods, following washing of cells, saponin lysis of red blood cells and digestion with proteinase K [21]. The DNA sample was resuspended in 30–50 μl of sterile TE buffer, and stored at -20°C until use.

### Polymerase chain reaction amplification of the *Pvmsp-3α *gene

Allelic diversity of the *msp-3α *gene was studied using the modified PCR-RFLP method described by Bruce and colleagues [13]. Briefly, the *msp-3α *gene was amplified by a nested PCR, using 2 μl of DNA extract in first reaction and 0.1 μl of the primary reaction in the second amplification. The oligonucleotide PCR primers were as described by Bruce and co-workers [13]. One unit of Taq polymerase, with 0.2 μM final concentration of both primers and 0.2 mM of each 4-deoxynucleotide triphosphates were used in reaction buffer containing 1.5 mM MgCl_2_. The reaction was carried out for 94°C for 3 min, 35 cycles at 94°C for 1 min, 56°C for 1 min, and 72°C for 2.5 min and a final primer extension at 72°C for 10 min. However, in second round reaction, 95°C for 2 min, 30 cycle at 94°C for 1 min, 62°C for 1 min, 72°C for 1.5 min and a final primer extension at 72°C. Two μl of the second PCR product was electrophoresed on a 1.5% agarose gel.

### PCR-RFLP analysis of *Pvmsp-3α *gene

For RFLP analysis, both *Hha *I and *Alu *I were used for digestion [13]. Approximately 10 μl of the PCR product was digested individually with the both restriction enzymes in total 20 μl reaction volumes as described by manufacture (Roche Company). The DNA fragments were visualized under UV illumination after electrophoresis on 2% agarose gels. Major alleles were classified based on the differences in restriction banding patterns.

## Results

### PCR amplification of the *Pvmsp-3α *gene

Fifty northern and 94 *P. vivax *southern isolates were successfully amplified for the *msp-3α *gene. A total of 99 distinct *Pvmsp*-3α fragments defined by the size were detected in the 94 southern samples by PCR analysis, showing that some patients (five of 94) had infections of more than one parasite genotype. No mixed genotype infections have been detected among northern isolates.

**Figure 1:**
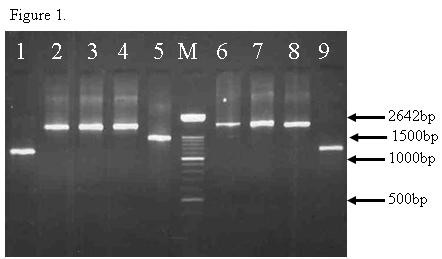
Figure 1: Lane 1-5 and 6-9 are uncut *msp-3α* polymerase chain reaction amplification products of some representative samples of Iranian P. *vivax* isolates. The lane with the molecular weight marker (100 bp ladder) is labeled M (Roche)

**Figure 2:**
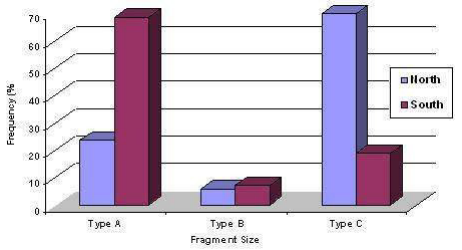
Frequency distribution of allele sizes, Type A (1.8 bp), Type B (1.5 bp) and Type C (1.2 bp) found in *P. vivax *isolates collected in the northern (n = 50) and southern isolates (n = 94) endemic areas.

Based on the length variants of the PCR products, three allele sizes, Type A (1.8 kb), Type B (1.5 kb) and Type C (1.2 kb) have been detected among both northern and southern isolates (Figure 1). The predominant fragments among northern and southern parasites were Type C (70%) and Type A (68.7%)  respectively.  However, the remaining of 24% (12 of 50) of northern isolates was Type A, and 6% (3 of 50) were Type B. In addition, 7% (7/99) of southern samples were Type B, and 19% (18/99) were Type C   (Figure 2).

### PCR-RFLP analysis of *Pvmsp-3α *gene

**Figure 3:**
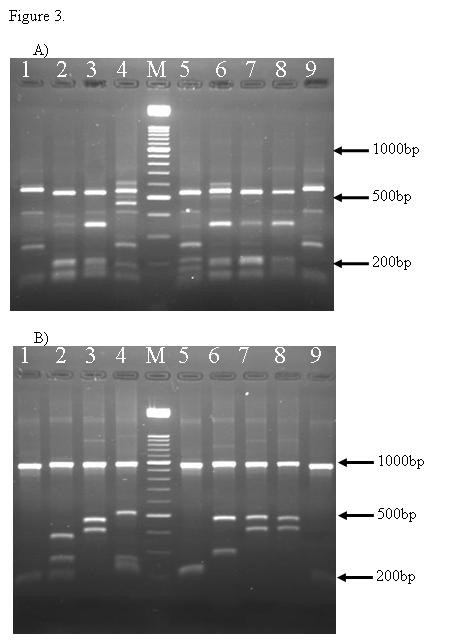
PCR-RPLF patterns of Iranian *P. vivax *isolates based on *Pvmsp-3α*. The amplification products were digested by Alu I (A) and Hha I (B). The lane with the molecular weight marker (100 bp ladder) is labeled M.

Both *Hha I *and *Alu I *enzymes showed clear restriction patterns in all analyzed samples. In all northern and southern isolates the RFLP patterns showed 500–600 bp for *Alu I *digests (Figure 3A) and the largest fragments between 950–1100 bp for *Hha I *(Figure 3B), as showed by others [13,22,23]. These fragments were not included for distinguishing different alleles, as it was difficult to resolve, however, smaller fragments from 150–750 bp were applied for RFLP analysis in this study (Figure 3).

Based on restriction patterns from digestion of PCR products with *Hha I *and *Alu I*, 12 and 49 distinct variants have been detected among 50 northern and 94 southern isolates.

As we have shown before, based *on msp-1 *analysis, 30 distinct variants identified in all 146-sequenced Iranian *P. vivax *isolate. These variants were grouped in; Type 1 (B1–B9), Type 2 (S1–S16) and Type 3 (R1–R5) [20]. However, by using *msp-3α *gene, followed by RFLP analysis with *Hha I *and *Alu I *enzymes, more variation between and within different Type and sub Types of Iranian *Pvmsp-1 *were detected. Based on *Pvmap-3α*, the most diverse group of *Pvmsp-1 *was Type 2 S1 variants with 10 allelic forms (Table 1). Furthermore, the number of different allelic variants of *Pvmsp-3α *in Type 1 (B1–B9), Type 2 (S1–S16), and Type 3 (R1–R5) of *Pvmsp-1 *has been shown in Table 1. The two samples belonging to Type 3 R2 were not included in this analysis because there were no more blood and DNA samples available at the time of this study. The RFLP results showed high diversity among southern isolates in comparispn to northern isolates that was similar to the results obtained previously [20].

**Table 1:** 

**Isolate Group**	**Amplified fragment size**			**Number of the allelic variant based on RFLP method**	
	
	**Type A(1800 bp)**	**Type B(1500 bp)**	**Type C(1200 bp)**	**Hha I**	**Alu I**
**B1N**	3/37 (8.1%)	2/37(5.4%)	31/37(83.8%)	4	4
**B1S**	-	-	1/37 (2.7%)	1	1
**B2N**	-	-	2/2 (100)	1	1
**B2S**	-	-	-	-	-
**B3N**	-	-	-	-	-
**B3S**	3/3 (100%)	-	-	2	2
**B4N**	-	-	-	-	-
**B4S**	1/1 (100%)	-	-	1	1
**B5N**	-	-	-	-	-
**B5S**	-	1/1 (100%)	-	1	1
**B6N**	-	-	-	-	-
**B6S**	2/2 (100%)	-	-	1	1
**B7N**	-	-	-	-	-
**B7S**	-	-	2/2 (100%)	2	2
**B8N**	-	-	-	-	-
**B8S**	3/3 (100%)	-	-	1	1
**B9N**	-	-	-	-	-
**B9S**	3/3 (100%)	-	-	2	2
**S1N**	5/23 (21.7%)	1/23 (4.3%)	-	3	3
**S1S**	13/23 (56.5%)	2/23 (8.7%)	2/23 (8.7%)	7	7
**S2N**	3/9 (34%)	-	1/9 (11%)	2	2
**S2S**	1/9 (11%)	2/9 (22%)	2/9 (22%)	3	3
**S3N**	-	-	1/1 (100%)	1	1
**S3S**	-	-	-	-	-
**S4N**	-	-	-	-	-
**S4S**	1/1 (100%)	-	-	1	1
**S5N**	-	-	-	-	-
**S5S**	2/2 (100%)	-	-	1	1
**S6N**	-	-	-	-	-
**S6S**	1/1 (100%)	-	-	1	1
**S7N**	-	-	-	-	-
**S7S**	1/1 (100%)	-	-	1	1
**S8N**	-	-	-	-	-
**S8S**	2/2 (100%)	-	-	1	1
**S9N**	-	-	-	-	-
**S9S**	5/5 (100%)	-	-	1	1
**S10N**	-	-	-	-	-
**S10S**	3/3 (100%)	-	-	2	2
**S11N**	-	-	-	-	-
**S11S**	1/1 (100%)	-	-	1	1
**S12N**	-	-	-	-	-
**S12S**	2/9 (22.2%)	1/9 (11.1%)	6/9 (66.7%)	2	2
**S13N**	-	-	-	-	-
**S13S**	1/2 (50%)	-	1/2 (50%)	2	2
**S14N**	-	-	-	-	-
**S14S**	1/1 (100%)	-	-	1	1
**S15N**	-	-	-	-	-
**S15S**	2/2 (100%)	-	-	2	2
**S16N**	-	-	-	-	-
**S16S**	1/1 (100%)	-	-	1	1
**R1N**	1/3 (33.4%)	-	-	1	1
**R1S**	2/3 (66.6%)	-	-	2	2
**R2N**	-	-	-	-	-
**R2S**	-	-	-	-	-
**R3N**	-	-	-	-	-
**R3S**	2/4 (50%)	1/4 (25%)	1/4 (25%)	3	3
**R4N**	-	-	-	-	-
**R4S**	2/2 (100%)	-	-	2	2
**R5N**	-	-	-	-	-
**R5S**	13/17 (76.5%)	-	4/17 (23.5%)	4	4

**Total (144)**	80 (55.5%)	10 (7%)	54 (37.5%)	61	61

## Discussion

The majority of researchers have used *msp-1 *gene as molecular markers for structural analysis of wild *P. vivax *isolates from different malaria endemic areas, which relies almost entirely on sequencing. However, the *Pvmsp-3α *gene has been recently applied and suggested as a polymorphic marker for molecular epidemiological studies [13,14]. This survey has been carried out to evaluate the *Pvmsp-3α *marker for molecular epidemiology studies of *P. vivax*. *Pvmsp-3α *was applied to the same DNA samples of *P. vivax *isolates from Iran that had been previously analysed with the *Pvmsp-1 *marker. The analysis of *Pvmsp-1 *gene in Iranian isolates had been carried out by sequencing analysis while the *Pvmsp-3α *gene was only analysed using the PCR-RFLP method.

The PCR-RFLP analysis of the *msp-3α *gene demonstrated that *P. vivax *parasites were highly diverse in Iran. Furthermore, the parasite population was more diverse among southern isolates compared to northern isolates, which was in concordance with the result of the *Pvmsp-1 *sequencing analysis. In addition, sequencing analysis of the *Pvmsp-1 *gene showed 9 different allelic variant of Type 1 (B1–B9), 16 allelic variant of Type 2 (S1–S16) and 5 allelic variants of Type 3 (R1–R5) in 146 isolates. Within the same type and allelic variant group, all samples showed 100% similarity at both nucleotides and protein levels [20]. However, in the present study using a *Pvmsp-3α *analysis of the same samples, the results showed greater variation within each group. Therefore, these results support that this genetic marker may be more useful and sensitive than the *msp-1 *marker, without the need for further sequencing analysis.

Furthermore, in the previous *msp-1 *study, [20] none of the samples showed any multiple infections, while 5 of 144 isolates showed multiple infections by using the *msp-3α *marker.

## Conclusion

The results demonstrated that the *Pvmsp-3α *is a powerful polymorphic marker, which could be applied for both genotyping, identification of mixed genotype parasite infections, and population structural analysis of *P. vivax *isolates. It is of particular importance to note that the *msp-3α *marker could be used with PCR-RFLP alone, without the need for further sequencing.

## Authors' contributions

S. Zakeri designed the study and was responsible for both supervision of laboratory work, development of the protocols, analysis of the data and writing up the paper. H. Barjesteh carried out the laboratory work. N. D. Djadid helped with data analysis and editing the manuscript. All authors read and approved the manuscript.
